# Foreign Body in the Eustachian Tube: A Challenging Diagnosis and Management

**DOI:** 10.4274/tao.2020.6058

**Published:** 2021-03-26

**Authors:** Catarina Rato, Gustavo Lopes, Delfim Duarte, Nuno Oliveira

**Affiliations:** 1 Department of Otorhinolaryngology & Head and Neck Surgery, Hospital Pedro Hispano, Matosinhos, Portugal

**Keywords:** Eustachian tube, foreign body, stapes surgery, endoscopy, middle ear, otosclerosis

## Abstract

Foreign bodies in the external ear are very common. The same cannot be said about foreign bodies in the Eustachian tube (ET). We report the case of a 63-year-old woman with a history of painless left side otorrhea and hearing loss. She reported a left ear surgery when she was 30-year-old but she did not know the diagnosis that was made at that time neither the kind of surgery performed. Otoscopic examination revealed an inferior perforation of the eardrum. Audiologic evaluation demonstrated a unilateral, moderate-severe mixed hearing loss. Computed tomography scan showed, in left ear, a soft tissue density filling the middle ear cavity and a foreign body in ET. The patient underwent middle ear exploration which required endoscopic assistance to visualize and remove the foreign body. It appeared to be a stapes prothesis of Robinson type. The displacement of a stapes prosthesis to the ET has not been reported in the literature. Surgeries in this region are challenging. This case highlights the importance of the integration of endoscopy into otologic surgery.

## Introduction

The Eustachian tube (ET) connects the middle ear to the nasopharynx (1). Its functions include ventilation, clearance and protection (1). Middle ear diseases may be due, at least in part, to failure or inadequacy of ET function (2). Sometimes the causes for this dysfunction are relatively easy to assess by history and physical examination (2). On the other hand, rare causes, such as ET obstruction by a foreign body, are only identifiable after imaging of temporal bone (3).

Minimal working space and compromised visual field make surgeries in the region of the ET challenging (3).

## Case Presentation

A 63-year-old woman presented with a history of painless left side otorrhea and hearing loss. She reported a left ear surgery when she was 30-year-old but she did not know the diagnosis that was made at that time neither the kind of surgery performed. At the age of 50 years old, the patient noticed an increasing hearing loss on the left side and ipsilateral intermittent otorrhea. Otoscopic examination revealed an inferior perforation of the eardrum with granulation tissue at the edges and inflamed middle ear mucosa. The opposite ear was normal.

**Figure 1:**
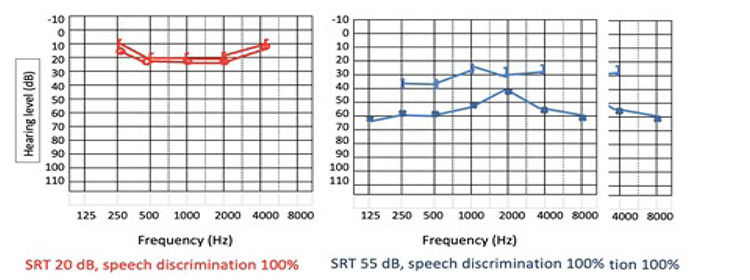
Audiogram before surgery SRT: Speech reception threshold

Pure tone audiometry demonstrated a moderate to severe left mixed hearing loss, with a 30 dB air-bone gap (Figure 1).

**Figure 2:**
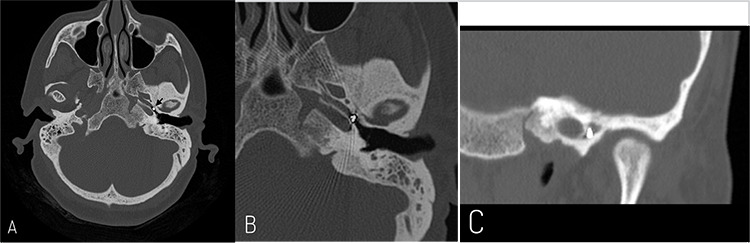
Computed tomography scan, temporal bones. Axial section (A) showing a foreign body in left Eustachian tube (arrow). Magnified axial (B) and coronal (C) sections demonstrating the proximity to the carotid artery

Computed tomography (CT) showed, in left ear, the presence of fluid and mucus in the mastoid air cells and a soft tissue density material occupying partially the middle ear cavity encasing the auditory ossicles without visible stapes. It also reported an incidental metallic foreign body with an intensity of 3,000 HU located at 3.5 mm from the ET opening in the middle ear and measuring 3.2x3.0 mm in size (Figure 2). In the right ear, there was also some fluid and mucus in the mastoid air cells but without any bone changes or associated ossicular chain abnormalities.

The diagnosis of foreign body in the ET associated with a chronic otitis media was made and surgical treatment was discussed with the patient, who consented a retroauricular exploratory tympanotomy.

**Figure 3:**
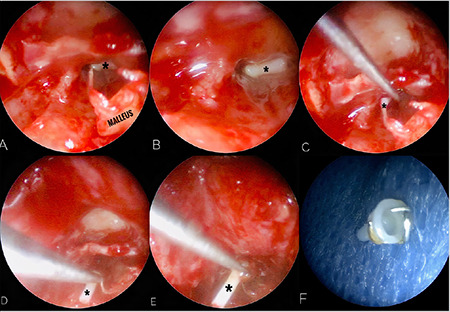
Intraoperative endoscopic findings. Only with endoscopic assistance was possible to see the foreign body (asterisk) (A and B). A fine curved pick was introduced into the left Eustachian tube orifice (C) and the foreign body was mobilized (C and D) and removed (E). It seemed to be a stapes prothesis of Robinson type made of metal wire and fluroplastic (F)

First, with microscopic approach, the tympanomeatal flap was elevated and the middle ear was explored. Absence of the stapes and eroded incus were observed. After many unsuccessful attempts with microscope, it was only possible to see the foreign body in the ET thanks to the use of a 4 mm 30^o^ endoscope (Figure 3A and 3B). The ET is not easily accessible with routine otological instruments. Removal of the foreign body was possible with the help of a fine curved pick (Figure 3C, 3D and 3E). It seemed to be a stapes prothesis of Robinson type made of metal wire and fluoroplastic (Figure 3F). The ET was found patent. A cartilage and perichondrium graft was used for tympanic membrane reconstruction.

The patient recovered well with resolution of chronic otitis media. After one year of follow-up, the tympanic membrane is intact and preoperative hearing level is stable. The options for management of hearing loss, revision stapes surgery or conventional hearing aids were considered with the patient. However, to this date, she deferred any further surgical intervention.

## Discussion

This case illustrates several interesting dilemmas. The similarity between the removed foreign body and a Robinson stapes prosthesis suggests that the surgery that patient underwent when she was 30-year-old was a stapes surgery. Probably, the displacement of the prothesis, followed by medial migration and obstruction of the ET, led to the development of chronic otitis media. However, there is no way to confirm the sequence of these events.

One of the most frequent complication of stapes surgery is the partial or total displacement of the prothesis (4). This finding has been correlated with erosion or fracture of incus long process (4). Nevertheless, in this case, it is also difficult to confirm if the incus erosion happened first or if was a consequence of chronic otitis media.

There are reports of stapes protheses extruded into the external ear canal (5). However, in this patient, there seems to have been a medial migration. Perhaps this can be explained by the clearance function of the ET. To our knowledge, this is the first report of a stapes prothesis dislocation to ET.

Another interesting aspect revolves around the difficulties of surgeries in ET region. Although endoscopic assistance was not initially planned, it was essential to visualize and remove the foreign body. Traditionally, with microscope approach, the presence of a foreign body in the ET require an extra dissection to increase the working space and vision (3, 6). Endoscopic ear surgery, with its wide field of view, permits to preserve the normal anatomy as much as possible and to complete more tasks transcanal (7), which may be, in the future, the preferred initial approach in similar cases.

There have been reports of foreign bodies in the ET causing erosion of the carotid canal (8). The surgeon must keep in mind that the internal carotid artery lurks closely behind the bony wall (1). Although it might be easier to see the foreign body with the help of the endoscope, it can be difficult to manipulate with conventional instruments. Already available angled endoscopic ear instruments might be useful (3). There are many cases in which the foreign body tends to become displaced deeper (3). In cases where it is not possible to remove the foreign body, Parelkar et al. (6) propose to leave it and fashion a graft which would ventilate the middle ear.

Despite the concern regarding the possibility of a scar in ET being itself also a cause for its dysfunction, in this case, the repair of tympanic membrane was attempted with success. The replacement of stapes prothesis was not performed at the same surgical time to reduce the bacterial content of middle ear before opening the vestibule.

## Conclusion

Foreign bodies can be one of the causes of ET dysfunction. Performing a CT scan may aid not only in surgical approach but also reveal unexpected and diagnostic findings. We report a very rare case of a stapes prothesis as an ET foreign body. Endoscopic, minimally invasive, and atraumatic techniques are recommended in surgeries in ET region.
